# Novel Bicyclic *P*,*S*-Heterocycles via Stereoselective *hetero*-Diels–Alder Reactions of Thiochalcones with 1-Phenyl-4*H*-phosphinin-4-one 1-Oxide †

**DOI:** 10.3390/molecules29092036

**Published:** 2024-04-28

**Authors:** Grzegorz Mlostoń, Katarzyna Urbaniak, Marcin Palusiak, Elżbieta Łastawiecka, Sławomir Frynas, Kazimierz Michał Pietrusiewicz, Heinz Heimgartner

**Affiliations:** 1 Department of Organic and Applied Chemistry, Faculty of Chemistry, University of Lodz, Tamka 12, 91-403 Lodz, Poland; katarzyna.urbaniak@chemia.uni.lodz.pl; 2 Department of Physical Chemistry, Faculty of Chemistry, University of Lodz, Pomorska 163/165, 90-236 Lodz, Poland; marcin.palusiak@chemia.uni.lodz.pl; 3 Department of Organic and Crystal Chemistry, Institute of Chemical Sciences, Faculty of Chemistry, Maria Curie-Skłodowska University, Gliniana 33, 20-614 Lublin, Poland; elzbieta.lastawiecka@mail.umcs.pl (E.Ł.); slawomir.frynaslas@mail.umcs.pl (S.F.); 4 Department of Chemistry, University of Zurich, Winterthurerstrasse 190, CH-8057 Zurich, Switzerland; heinz.heimgartner@chem.uzh.ch

**Keywords:** *hetero*-Diels–Alder reaction, thiochalcones, *P*-heterocycles, *S*-heterocycles, fused *P*,*S*-heterocycles

## Abstract

Thiochalcones undergo cycloaddition reactions in THF solution at 60 °C with the synthetically unexplored 1-phenyl-4*H*-phosphinin-4-one 1-oxide in a highly regio- and stereoselective manner, yielding hitherto unknown bicyclic *P*,*S*-heterocycles containing fused thiopyran and phosphinine rings. The stereochemical structures of two of the obtained (4+2)-cycloadducts were unambiguously assigned by means of the X-ray single-crystal analysis. Based on these assignments, a concerted mechanism of the *hetero*-Diels–Alder reaction via the preferred *endo* approach of the heterodiene from the less hindered P=O side of the phosphininone molecule is postulated to explain the established rac-(4*RS*,8*SR*,9*SR*,10*SR*)-configured (4+2)-cycloadducts isolated as major products.

## 1. Introduction

**Figure 1:**
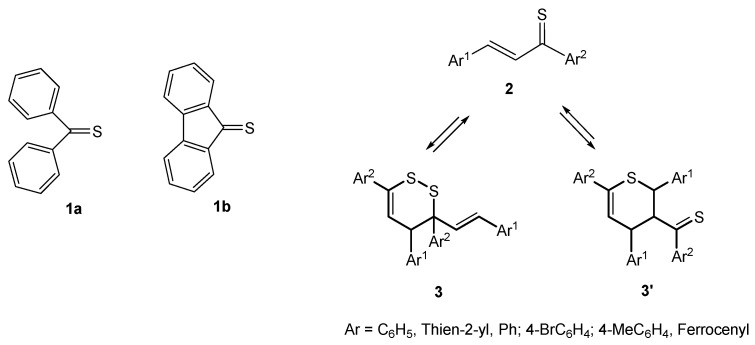
Aromatic thioketones **1a** and **1b** and the equilibrium of the monomeric (**2**) and dimeric (**3**/**3′**) structures of thiochalcones in solutions [6,9].

Aromatic thioketones such as thiofluorenone (**1a**) and thiobenzophenone (**1b**) are recognized as superior reagents for trapping 1,3-dipoles and dienes, hence termed as ‘superdipolarophiles’ (Rolf Huisgen 1995) [1,2,3] and ‘superdienophiles’ (Jürgen Sauer 1998) [4,5], respectively. The α,β-unsaturated analogs of aromatic thioketone **1**, known as thio-chalcones, exist in solution as a mixture of the monomeric **2** and two dimeric forms, **3** and **3′** (Figure 1). In general, contrary to chalcones, they have been less explored in the current organic synthesis, particularly in the cycloaddition chemistry (e.g., [6,7,8]). 

**Scheme 1:**
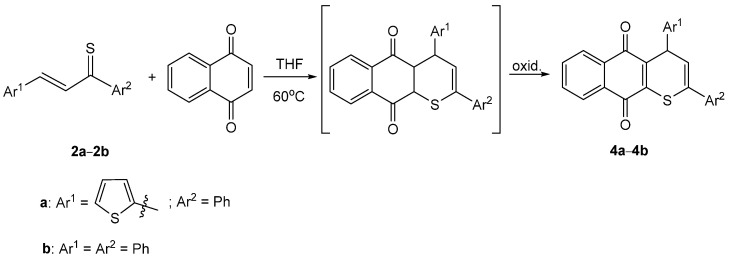
Recently described *hetero*-Diels–Alder reactions of thiochalcone **2** with naphthoquinone, leading to polycyclic *S*-heterocycle **4** [15].

However, in our recent publications, diverse thiochalcones (as monomeric forms **2**) bearing aryl, hetaryl, or ferrocenyl groups have been described for the first time as active dipolarophiles in (3+2)-cycloadditions with electron-deficient, fluorinated nitrile imines [9] and as dienophiles in (4+2)-cycloadditions with electron-deficient α-nitroso alkenes [10]. Additionally, thiochalcones were successfully applied as dienophiles or *S*-heterodienes in some asymmetric *hetero*-Diels–Alder reactions [11,12]. Moreover, they smoothly undergo (4+2)-cycloaddition reactions with acetylenic carboxylates [13,14] and some 1,4-quinones, e.g., with naphthoquinone to give fused 4*H*-thiopyran **4** in high yields after spontaneous oxidation of the initial (4+2)-cycloadducts [15] (Scheme 1). 

**Figure 2:**
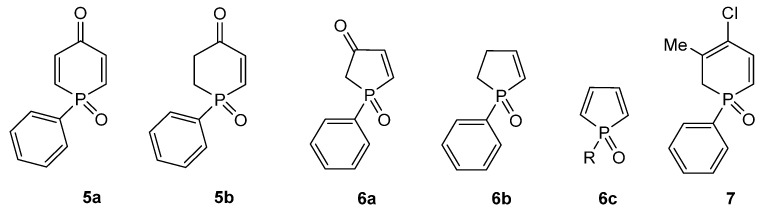
The ‘quinone like’ *P*-heterocycles **5a** and **5b**, the five-membered phosphole 1-oxide derivatives **6a**–**6c**, and dihydrophosphinine 1-oxide **7** as potential or already studied dienophiles to be used in (4+2)- and (3+2)-cycloaddition reactions.

**Scheme 2:**
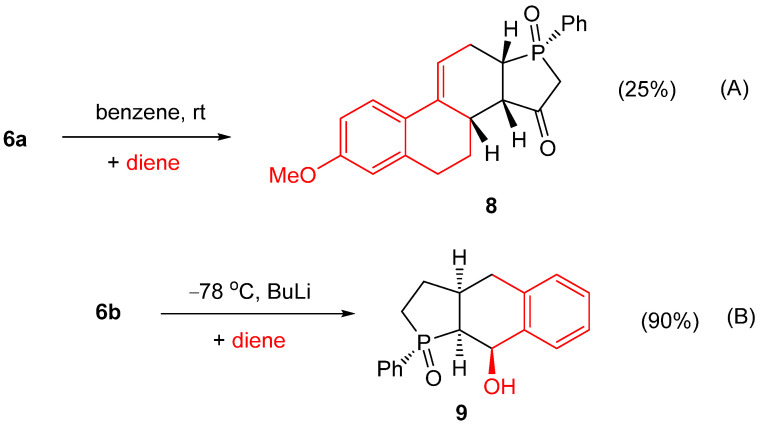
Reported examples of stereoselective Diels–Alder reactions with phospholene 1-oxides **6a** and **6b**, leading to 15-*P*-steroid **8** (ref. [19]) and the fused *P*-heterocycle **9** (ref. [20]), respectively. The corresponding diene framework in cycloadducts **8** and **9** are presented in red.

Many years ago, a rare six-membered phosphorus heterocycle (4*H*-phosphinin-4-one) was reported as a stable 1-oxide **5a** [16]. In recent publications, the synthesis of its dihydro derivative **5b** was also described [17,18] (Figure 2). The structures of both compounds resemble the 1,4-quinone framework, strongly suggesting their potential reactivity toward dienes/heterodienes and, thus, a possible application for the synthesis of new, fused *P*-heterocyclic systems. This expectation is reinforced by the fact that five-membered ring congeners of phosphinine 1-oxides **5a** and **5b**, namely phospholene 1-oxide **6a** and its derivatives **6b** and **6c**, as well as the dihydrophosphinine 1-oxide **7**, have already been reported to act as dieno- and dipolarophiles in the cycloaddition chemistry (Scheme 2). 

**Scheme 3:**
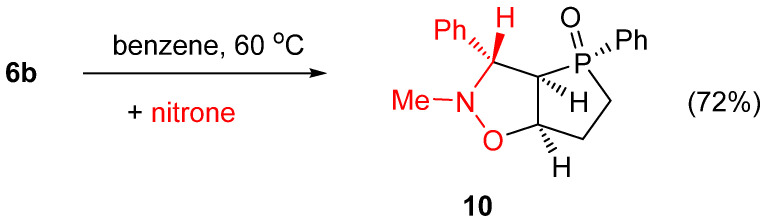
Reported example of a stereoselective (3+2)-cycloaddition of phospholene 1-oxide **6b** with a nitrone acting as a 1,3-dipole (ref. [21,22]). The nitrone framework in cycloadduct **10** is presented in red.

**Scheme 4:**
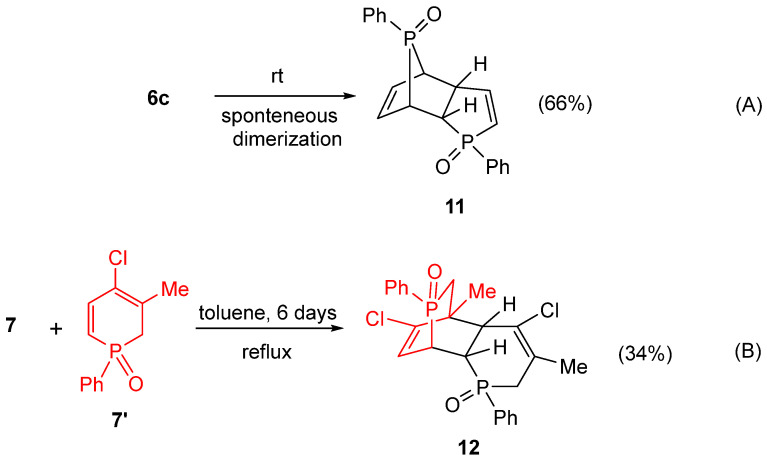
Stereoselective dimerization of phospholene 1-oxide **6c** and phosphinine 1-oxide **7** leading to polycyclic *P*-heterocycles **11** and **12**, respectively (ref. [23,24] and [25]). The diene molecule **7′** and diene framework in cycloadduct **12** are presented in red.

Cycloaddition reactions involving the exploration of *C*,*P*-heterocycle 1-oxides as dieno- or dipolarophiles are rarely described. Representative examples of such reactions, leading to a variety of polycylic *P*-heterocycles, as illustrated in Scheme 2, Scheme 3 and Scheme 4, have already been reported.

As shown in Scheme 2, the highly activated phospholene 1-oxide **6a** easily enters the Diels–Alder reaction with 7-methoxy-4-vinyl-1,2-dihydronaphthalene (diene), stereoselectively yielding the tetracyclic cycloadduct **8** with a 15-*P*-steroid structure in 25% yield (Scheme 2A) [19]. Similarly, phospholene 1-oxide **6b** was reported to undergo the Diels–Alder reaction with in situ-generated *α*-oxy-*o*-xylylene (diene), leading to the tricyclic *P*-heterocycle **9** in 90% yield (Scheme 2B) [20].

The same substrate **6b** was successfully employed as a dipolarophile in the (3+2)-cycloaddition with an acyclic nitrone (*C*-phenyl-*N*-methylnitrone), yielding the P-containing fused heterocycle **10** in 72% yield with high stereoselectivity (Scheme 3) [21,22].

Furthermore, the phospholene 1-oxide **6c** undergoes dimerization according to the rules of (4+2)-cycloadditions, yielding the tricyclic *P*-heterocycle **11** in suitable yield (66%) with high stereoselectivity [23,24] (Scheme 4A). 

The only known example of a (4+2)-cycloaddition involving the six-membered phosphinine 1-oxide **7** is depicted in Scheme 4B. However, in this instance, the observed dimerization-like cycloaddition did not occur between two molecules of **7**. Instead, **7** was trapped by its double bond isomer **7′** present in the reaction mixture, leading to the stereo-selective formation of the tricyclic product **12** (Scheme 4B) [25]. This unexpected reaction provides an intriguing example of so-called cross-dimerizations. 

The objective of the present study was to investigate the reactivity of 4*H*-phosphinin-4-one 1-oxides **5a** and **5b** toward selected thiochalcone **2**, which were employed as reactive heterodienes. Specifically, we aimed to elucidate the regiochemistry of the anticipated *hetero*-Diels–Alder reactions and the stereochemical structure of the resulting (4+2)-cycloadducts containing two heteroatoms (P and S). Additionally, the investigation of the reaction mechanism was of particular interest. 

## 2. Results and Discussion

The test experiment was carried out in THF solution at 60 °C using 1-phenyl-3-(thien-2-yl)propene-1-thione (**2a**) and 1-phenylphosphinin-4-one 1-oxide (**5a**) in a 1:1 ratio. TLC analysis revealed complete consumption of **2a** after approximately 20 h. After the removal of THF in vacuum, the crude reaction mixture was analyzed by ^1^H NMR, indicating the presence of two products formed in unequal amounts. The major product exhibited three signals in the non-aromatic region at 3.63–3.66, 4.03–4.05, and 4.54–4.57 ppm, which can be attributed to aliphatic protons. Additionally, three similar but less intense signals were observed for the minor product at 3.69‒3.71, 4.06–4.08, and 4.49–4.50 ppm, respectively (Supplementary Figure S1). The close resemblance of the corresponding signals of the major and minor products strongly suggested that they shared the same framework with identical positions of both heteroatoms (S and P), differing most likely only in the relative stereochemistry at stereogenic centers. A comparison of the intensities of the integration lines of multiplets attributed to the major and minor components suggested a ca. 7:1 ratio of these isomeric cycloadducts.

**Scheme 5:**
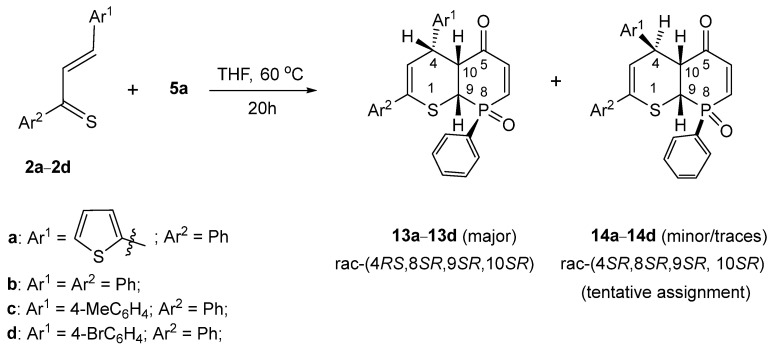
Diastereoselective *hetero*-Diels–Alder reactions of **5a** with thiochalcones **2a**–**2d** leading to fused *P*,*S*-heterocycles rac-(4*RS*,8*SR*,9*SR*,10*SR*)-**13a**–**13d** as major products and rac-(4*SR*,8*SR*,9*SR*,10*SR*)-**14a**‒**14d** (tentative assignment of configuration) formed in low yields.

**Figure 3:**
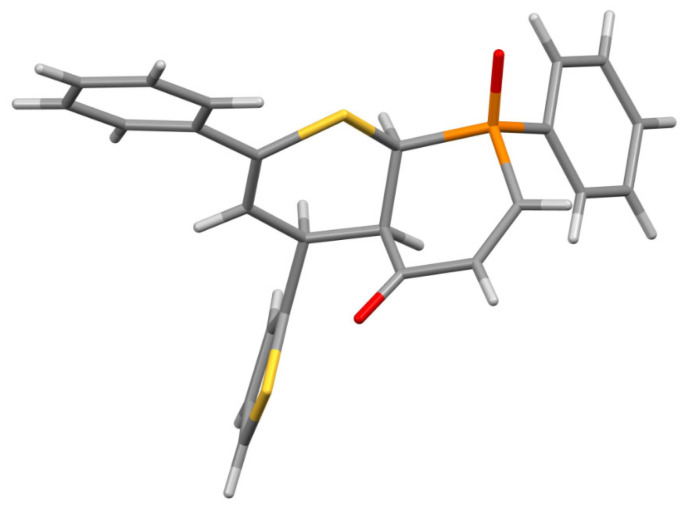
Molecular structure of the 4-(thien-2-yl)-substituted cycloadduct **13a**. For graphics with atoms represented by thermal ellipsoids (50%), see Figure S14.

Chromatographic separation of the crude reaction mixture on a SiO_2_ column afforded the major product with a melting point of 184 °C (decomposition) in a yield of 57%, using a petroleum ether/dichloromethane mixture as the eluent. The ^31^P NMR spectrum showed a resonance signal at 20.9 ppm for the P atom. The HRMS spectrum of this product revealed a molecular mass [M + 1] = 435.0642, corresponding to the molecular formula C_24_H_20_O_2_PS_2_, indicative of the (4+2)-cycloadduct of **2a** and **5a**. Finally, single-crystal X-ray diffraction analysis confirmed the structure of the fused *P*,*S*-heterocycle **13a**, with the heteroatoms located at S(1) and P(8) positions, respectively (for conventional atom numbering, see Scheme 5). In contrast to quinones (Scheme 1), no spontaneous oxidation of the initially formed cycloadducts was observed. Based on the X-ray analysis, the stereochemical structure of this diastereoisomer was determined as rac-(4*RS*,8*SR*,9*SR*,10*SR*) (Scheme 5 and Figure 3).

The aforementioned three sets of signals of the minor product could tentatively be attributed to an isomeric cycloadduct **14a** with rac-(4*SR*,8*SR*,9*SR*,10*SR*) configuration (Scheme 5). However, attempts to isolate this product by column chromatography were unsuccessful. 

**Figure 4:**
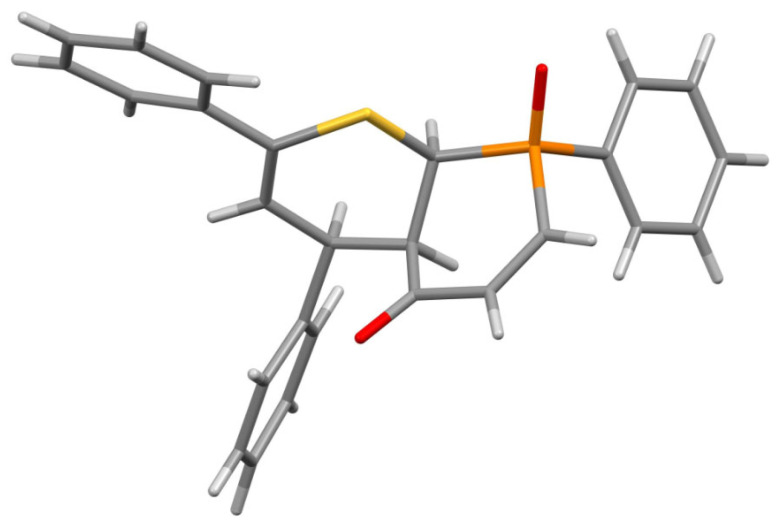
Molecular structure of the 4-phenyl-substituted cycloadduct **13b**. For graphics with atoms represented by thermal ellipsoids (50%), see Figure S15.

Reactions of thiochalcones **2b**–**2d** with **5a** (molar ratio 1:1) were carried out under analogous conditions to those described for **2a**. In all cases, as in the experiment with **2a**, the ^1^H NMR spectra obtained for the crude reaction mixtures revealed the presence of two isomeric cycloadducts of types **13** and **14**, exhibiting similar sets of signals in the previously described high-field regions of the ^1^H NMR spectra. However, in all cases, the yields of the postulated cycloadducts **14b**–**14d** were consistently very low (<10%), and their attempted isolation was again unsuccessful. The major product **13b**, bearing two phenyl substituents, was isolated chromatographically in 51% yield. Subsequent crystallization from petroleum ether/CH_2_Cl_2_ yielded single crystals suitable for the X-ray analysis. The analysis confirmed both the regiochemical structure of **13b** and the configurations of the four stereogenic centers at C(4), P(8), C(9), and C(10), indicating again the *endo* mode of addition in full analogy to **13a** (Scheme 5 and Figure 4).

**Scheme 6:**
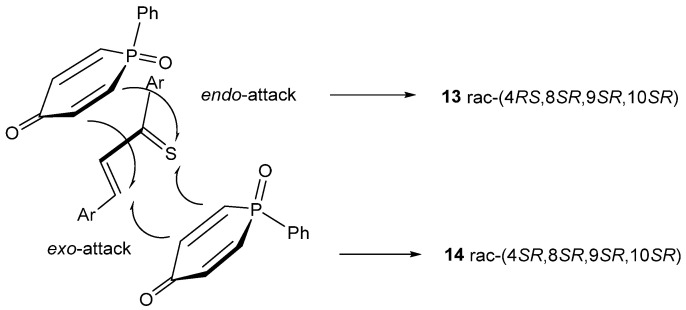
Proposed structure of the transition states of the (4+2)-cycloaddition of thiochalcone **2** with 1-phenyl-4*H*-phosphinin-4-one 1-oxide (**5a**).

It is worth mentioning that in separate NMR experiments, the stereochemical stability of **13b** was tested in CDCl_3_ solution at room temperature and at 60 °C, and in both cases, unchanged **13b** was observed thereafter. Moreover, addition of catalytic amounts of either TFA or pyridine to the sample did not result in any detectable isomerization of **13b,** neither at room temperature nor after heating at 60 °C. The observed lack of isomerization thus allowed us to exclude the possibility that the obtained minor products were formed via keto-enol equilibration of the corresponding major products under the reaction conditions. Considering the complete face selectivity recorded thus far for all known cycloadditions of cyclic organophosphorus dienophiles, which are exclusively attacked by a diene from their P=O bearing side (cf. Scheme 5 and Scheme 6), it became most plausible that the stereochemistry of the minor product **14** reflects the *exo* mode of their formation, as tentatively assigned (cf. Scheme 5).

Notably, the attempted *hetero*-Diels–Alder reaction of **5a** with the isomeric thiochalcone **2′a** (3-phenyl-1-(thien-2-yl)propene) in THF at 60 °C was unsuccessful, resulting in the decomposition of the starting materials. Apparently, the substitution pattern in the starting thiochalcone also influences the outcome of the studied reactions. 

Similarly, the attempted (4+2)-cycloaddition of thiochalcone **2b** with 2,3-dihydro-4*H*-phosphinin-4-one 1-oxide **5b** was unsuccessful, and no defined product could be identified in the crude reaction mixtures. 

*Structure analysis of cycloadducts* **13a** *and* **13b**: In our research endeavor, we successfully acquired crystal samples of compounds **13a** and **13b**, which proved to be suitable for conducting X-ray single-crystal experiments. Both compounds exhibit crystal structures within the centrosymmetric P2_1_/c space group. Consequently, the crystal samples applied in the analysis contain racemic mixtures of inversion-equivalent enantiomeric forms, aligning with the anticipated outcome within the context of our synthesis procedure. For a detailed insight into the absolute configuration of all four chiral centers in molecules of **13a** and **13b**, refer to Scheme 5. Furthermore, our crystallographic analysis revealed the presence of two molecules within the symmetry-independent cell unit in both crystals. There is no pseudo-symmetry between the molecules in the asymmetric unit. Each molecule possesses its own structural characterization in both experimentally treated samples.

Remarkably, these molecules within the symmetry-independent unit display identical absolute configurations.

The molecular architecture of both compounds is characterized by two fused heterocyclic rings, as depicted in Figure 3 and Figure 4. Notably, the C(9) and C(10) centers serve as common elements in both heterocycles. The hydrogen atoms attached to these carbon atoms adopt a *cis* configuration, leading to a distinctive nonplanar geometry of this primary molecular moiety and, consequently, influencing the overall molecular structure. This nonplanarity is reflected in the formation of dihedral angles of 79.74° and 75.16° for compounds **13a** and **13b**, respectively. In both crystal samples, the heterocyclic rings adopt a half-boat conformation, with the C(9) and C(10) atoms positioned above the mean plane of the rings. The distance between the carbon atoms at the apex position and the planar fragments of the rings is in the range of 0.654(3)–0.756(3) Å. Importantly, the heteroatoms S and P are always in the plane of their parent rings.

The planarity of other rings conforms to expectations, with their positioning relative to the bearing fragment determined by crystal packing, exhibiting no anomalous behavior. 

An intriguing observation arises from the absence of classic H-bond-donating groups in both molecules, thereby precluding the presence of traditional H-bonds (e.g., O–H**^…^**O or N–H**^…^**O type) responsible for stabilizing the crystal lattice. Instead, we identified numerous weak short contacts of C–H**^…^**A (where A = S and O), potentially serving as stabilizing interactions. Notably, our attention is particularly drawn to a short contact of P=O**^…^**C_sp_^2^ type, with O**^…^**C distances of 2.994(2) Å and 3.006(2) Å for compounds **13a** and **13b**, respectively. The corresponding P=O**^…^**C angles are measured to be 130.6(1)° and 137.5(1)°. Importantly, the involved carbon atoms belong to strongly polarized CO carbonyl groups, suggesting that these interactions may rival hydrogen bonding in terms of strength. As already postulated [26], such interactions exhibit a comparable strength to hydrogen bonding and are notably directional due to the partial atomic charge distribution within the interacting fragments. We are expecting that this specific P=O**^…^**C interaction may be a leading one among all, which stabilizes crystals of **13a** and **13b**.

*Mechanistic consideration*: The (4+2)-cycloadditions of electron-rich thiochalcone **2** with the phosphorus-containing dienophile **5a**, considered as an electron-deficient dienophile, are reported for the first time. Therefore, the mechanism of these *hetero*-Diels–Alder reactions depicted in Scheme 6 deserves a brief comment.

In recent decades, mechanisms of cycloaddition reactions have been studied intensively. Alongside the ‘classical’ interpretation based on the assumption of concerted pathways, stepwise processes via zwitterionic or diradical intermediates have also been discussed [27,28,29,30]. It is well known that the large energy gap between HOMO and LUMO energies of both reactants, i.e., 1,3-dipole and dipolarophile in the case of (3+2)-cycloadditions [2], and diene and dienophile in the case of Diels–Alder reactions [31], favors stepwise mechanisms. 

In our opinion, the studied reactions follow the classical concerted (but asynchronous) mechanism, and the preferred *endo*-attack of the heterodiene from the less hindered P=O side (face selectivity) of the organophosphorus compound leads to the isolated major cycloadduct rac-(4*RS*,8*SR*,9*SR*,10*SR*)-**13** (Scheme 6). The reactions occur with complete regioselectivity and lead to the formation of the C–S bond exclusively at the *β*-C atom. Moreover, it has already been well documented that the *α,β*-unsaturated *P*-heterocyclic dienophiles undergo cycloaddition reactions with 1,3-dipoles [21,22] as well as with dienes [19,20] via the approach from the P=O bearing face, and this spatial preference is observed for both the *endo* and the *exo* mode of addition [21,22]. The alternative *exo* attack results in the formation of (4+2)-cycloadduct **14** with an inverted configuration at the C(4) atom (Scheme 6).

## 3. Materials and Methods

### 3.1. Experimental: X-ray Structure Determination of ***13a*** and ***13b***

X-ray diffraction data for **13a** and **13b** were collected on an XtaLAB Synergy, Dualflex, HyPix diffractometer (Rigaku, Akishima, Japan). Integration of the intensities and corrections for Lorentz effects, polarization effects, and analytical absorption were performed with CrysAlis PRO [32]. Using Olex2 [33], the structures were solved with the SHELXT [34] structure solution program using Intrinsic Phasing and refined with the SHELXL [35] refinement package using Least Squares minimization. The hydrogen atoms were introduced in the calculated positions with an idealized geometry and constrained using a rigid body model with isotropic displacement parameters equal to 1.2 of the equivalent displacement parameters of their parent atoms. The molecular geometries were calculated by the PLATON program [36]. The relevant crystallographic data are provided in Table S1. Atomic coordinates, displacement parameters, and structural factors of the analyzed crystal structures are deposited with the Cambridge Crystallographic Data Centre CCDC (reference numbers: 2298978 and 2298979) [37] (for further details, see Supplementary Materials).

### 3.2. General Information

Commercial chemicals and solvents were used as received. If not stated otherwise, products were purified by filtration through short silica gel plugs (200–400 mesh) by using freshly distilled solvents as eluents or by recrystallization. Melting points were determined in capillaries with an Aldrich Melt-Temp II (St. Louis, MO, USA), and they are uncorrected. NMR spectra were taken with a Bruker AVIII spectrometer (Billerica, MA, USA, ^1^H NMR (600 MHz), ^13^C NMR (151 MHz), and ^31^P NMR (243 MHz)); chemical shifts (δ) are expressed in parts per million, and they relate to residual undeuterated solvent peaks (CDCl_3_: ^1^H NMR *δ* = 7.26, ^13^C NMR δ = 77.16) or to an external standard (H_3_PO_4_: ^31^P NMR δ = 0.00). IR spectra are presented in cm^‒1^, and they were measured with an Agilent Cary 630 FTIR spectrometer (Santa Clara, CA, USA) in neat. Mass spectra (ESI) were registered with a Varian 500-MS LC Ion Trap (Palo Alto, CA, USA). Elemental analyses were obtained with a Vario EL III (Elementar Analysensysteme GmbH, Langenselbold, Germany) instrument.

**Starting materials:** Thiochalcones **2a**–**2d** were prepared by treatment of the corresponding chalcones [38,39] with Lawesson’s reagent in boiling THF for 2.5–3 h. Crude products were purified by column chromatography and used for the studied reactions as unseparated mixtures of monomeric and dimeric forms **2**/**3**/**3′** [9,13]. 1-Phenyl-4*H*-phosphinin-4-one 1-oxide (**5a**) and 1-phenyl-2,3-dihydro-4*H*-phosphinin-4-one 1-oxide (**5b**) were synthesized following the published procedures [17,18].

**General procedure for reactions of thiochalcones 2a**–**2d with 4*H*-phosphinin-4-one 1-oxide 5a**: A solution containing 1.1 mmol of the corresponding thiochalcone **2** and 1 mmol of 4*H*-phosphinin-4-one 1-oxide **5a** dissolved in 4 mL of dry THF was heated in an oil bath at 60 °C overnight (about 20 h). After this time, the TLC test showed that starting **5a** was completely consumed. The solvent was evaporated, and the remaining mixture, after the ^1^H NMR examination, was purified by column chromatography (petroleum ether with increasing amounts of CH_2_Cl_2_). Analytically pure products were obtained by crystallization from petroleum ether/CH_2_Cl_2_.

**Figure 5:**
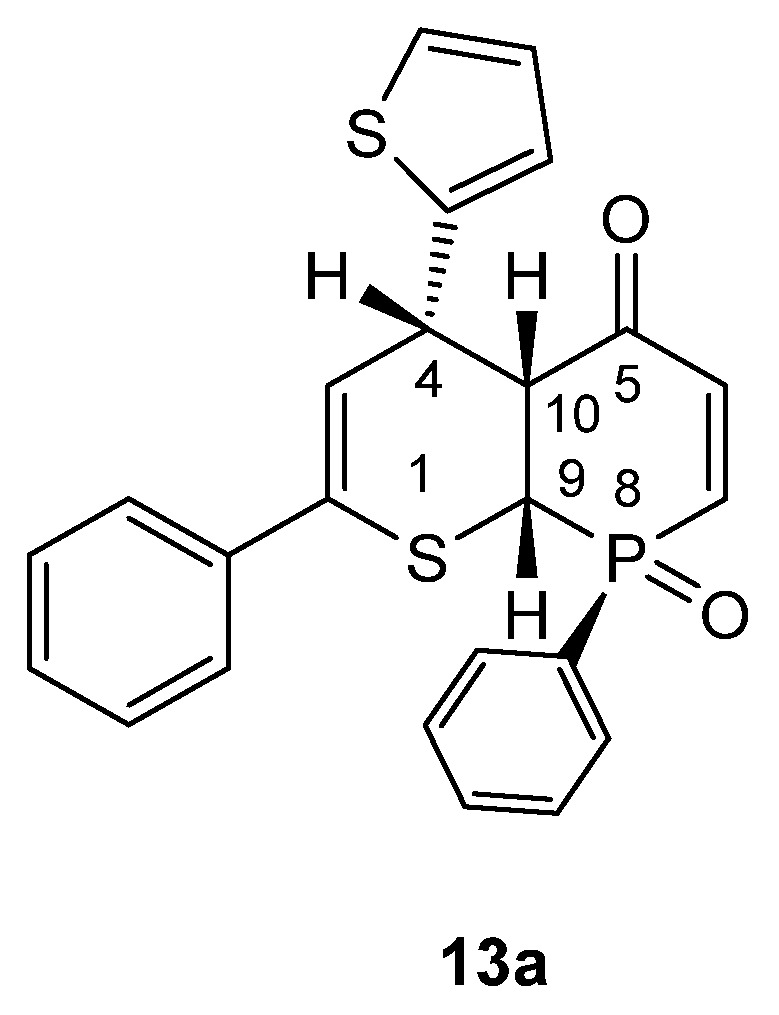
Structure of compound **13a**.

**Figure 6:**
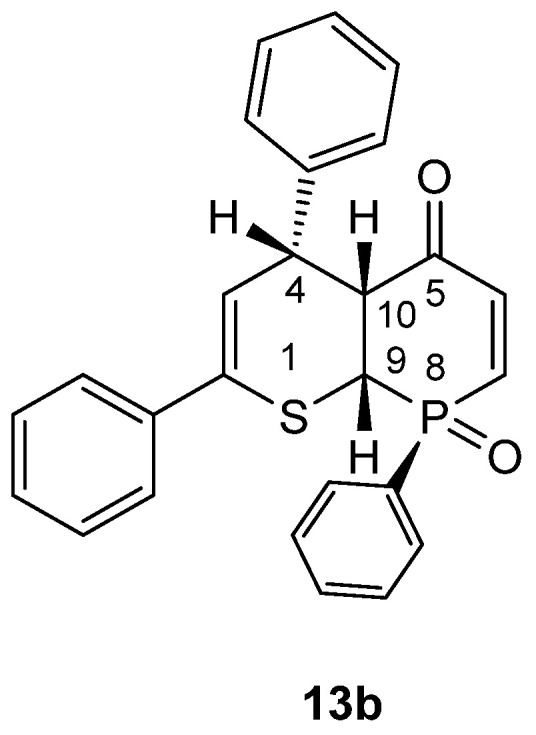
Structure of compound **13b**.

**Figure 7:**
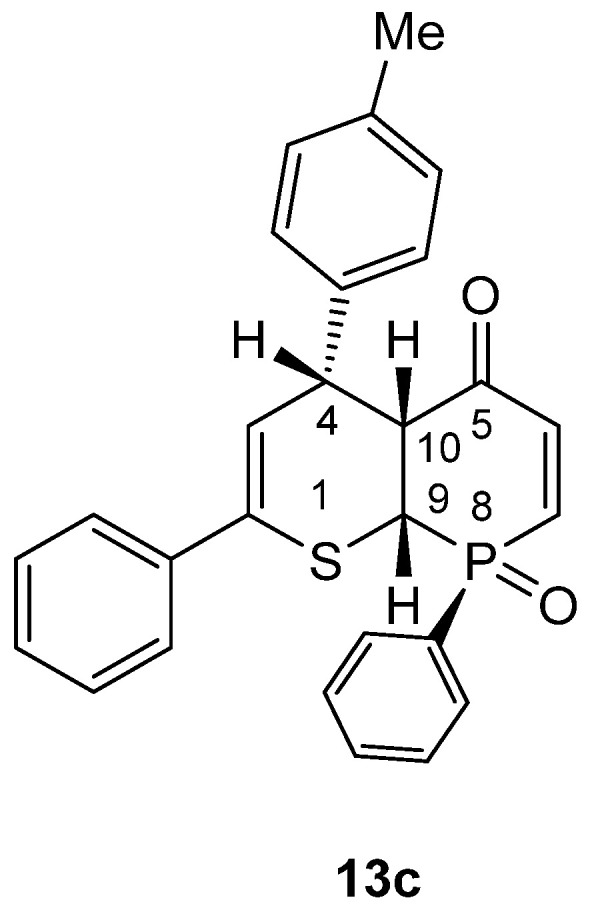
Structure of compound **13c**.

**Figure 8:**
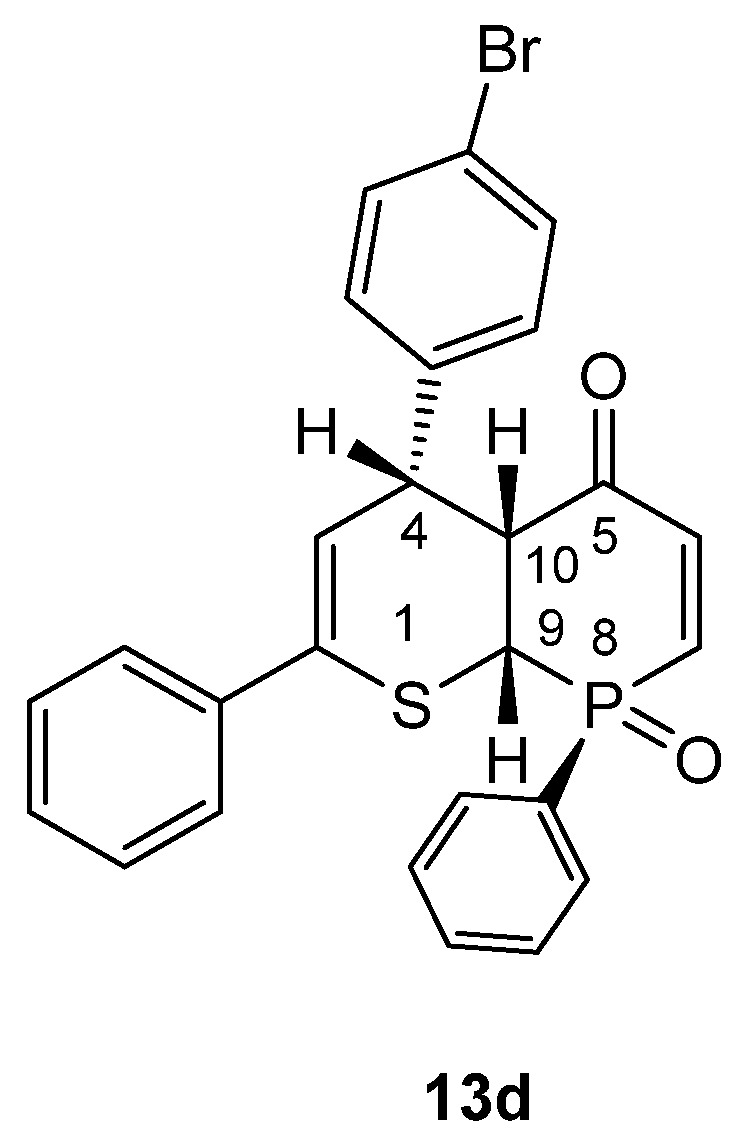
Structure of compound **13d**.

Structures of the isolated cycloadducts are presented in Figure 5, Figure 6, Figure 7 and Figure 8.

*rac-RS,8SR,9SR,10SR)-2,8-Diphenyl-*4*-(thiophen-2-yl)-4,4a,8a-trihydrophosphinino [2,3-b]thiopyran-5-one 8-oxide* (**13a**). Yield = 250 mg (57%), pale yellow crystals, mp = 184 °C (decomp.) (petroleum ether/CH_2_Cl_2_).

^1^H NMR (CDCl_3_): δ 3.63–3.66 (*m*, 1CH); 4.03 (*brs*, 1CH); 4.55 (*td*, *J* = 15.6 Hz, *J* = 2.7 Hz, 1CH); 6.36 (*d*, *J* = 2.4 Hz, 1CH); 6.77 (*dd*, *J* = 37.1 Hz, *J* = 12.9 Hz, 1CH); 6.87‒6.92 (*m*, 1CH); 6.97 (*dd*, *J* = 5.1 Hz, *J* = 3.5 Hz, 1CH); 7.04 (*d*, *J* = 3.5 Hz, CH); 7.22 (*dd*, *J* = 5.1 Hz, *J* = 1.0 Hz, 1CH); 7.33‒7.38 (*m*, 3CH_arom_); 7.51–7.54 (*m*, 2CH_arom_); 7.60–7.68 (*m*, 2CH); 7.75 (*dt*, *J* = 7.4 Hz, *J* = 1.2 Hz, 1CH); 7.92–7.96 (*m*, 2CH).

^13^C{^1^H} NMR (CDCl_3_): δ 41.2 (*J_C,P_* = 10 Hz), 48.6 (*J_C,P_* = 4 Hz) (2*d*, *C*(4) and *C*(10)—assignment uncertain); 44.5 (*d*, ^1^*J_C,P_* = 66 Hz, *C*(9)); 120.8, 124.6, 126.7, 128.5, 128.7, 130.9, 131.5, 138.4, 143.0 (9 signals), 126.3, (*d*, ^1^*J*_C,P_ = 47 Hz, *C*(7)), 129.7 (*d*, *J_C,P_* = 15 Hz), 131.1 (*d*, *J_C,P_* = 2 Hz), 133.8 (*d*, *J_C,P_* = 2 Hz), 128.5, (*d*, ^1^*J*_C,P_ = 105 Hz, *C*_ar_), 132.0 (*d*, *J* = 7 Hz); 144.2 (*d*, *J* = 2 Hz), 191.4 (*d*, ^3^*J_C,P_* = 11 Hz, *C*=O).

^31^P NMR (CDCl_3_): δ 20.9.

IR: 3024*m*, 3005*m*, 1694*s* (C=O), 1437*s*, 1239*m*, 1187*m*, 1168*s*, 1112*m*, 830*m*, 711*s*, 693*vs*.

HRMS for [M + 1]^+^ [C_24_H_20_O_2_PS_2_]: calc.: 435.0642; found: 435.0646.

EA for C_24_H_19_O_2_PS_2_ (434.51): calc. C 66.34, H 4.41, S 14.76 found C 66.09, H 4.54, S 14.98.

*rac-(RS,8SR,9SR,10SR)-2,4,8-Triphenyl-4,4a,8a-trihydrophosphinino[2,3-b]thiopyran-5-one 8-oxide* (**13b**). Yield: 220 mg (51%), beige crystals, mp = 212 °C (decomp.) (petroleum ether/CH_2_Cl_2_).

^1^H NMR (CDCl_3_): δ 3.63–3.66 (*m*, 1H); 3.78 (*brs*, 1H); 4.57–4.61 (*m*, 1H); 6.46 (*d*, *J* = 2.2 Hz, 1H); 6.69 (*dd*, *J* = 37.4 Hz*, J*_H,H_ = 13.0 Hz, 1H); 6.86–6.89 (*m*, 1H); 7.25–7.27 (*m*, 1H); 7.34–7.40 (*m*, 5H); 7.43–7.44 (*m*, 2H); 7.57–7.59 (*m*, 2H); 7.65–7.68 (*m*, 2H); 7.74–7.76 (*m*, 1H); 7.92–7.96 (*m*, 2H).

^13^C{^1^H} NMR (CDCl_3_): δ 44.9 (*d*, ^1^*J*_C,P_ = 73 Hz, *C*(9)), 45.5 (*J*_C,P_ = 2 Hz), 48.5 (*J*_C,P_ = 5 Hz) (2*d*, *C*(4) and *C*(10)—assignment uncertain); 120.5, 126.7, 126.9, 128.1, 128.5, 128.6 129.2, 138.8, 143.3 (9 signals); 128.6 (*d*, ^1^*J*_C,P_ = 104 Hz, *C*_ar_), 129.6 (*d*, ^2^*J*_C,P_ = 15 Hz), 131.05 (*d*, ^2^*J*_C,P_ = 10 Hz, =*C*(6)), 131.1 (*d*, ^1^*J*_C,P_ = 87 Hz, *C*(7)), 132.6 (*d*, *J*_C,P_ = 7 Hz), 133.7 (*d*, *J*_C,P_ = 2 Hz), 140.7 (*d*, *J*_C,P_ = 2 Hz); 191.8 (*d*, ^3^*J_C,P_* = 10 Hz, *C*=O).

^31^P NMR (CDCl_3_): δ 21.1.

IR: 2997*m*, 2893*m*, 1653*s* (C=O), 1567*m*, 1495*m*, 1432*m*, 1123*vs*, 1098*m*, 767*vs*, 698*vs*, 513s.

HRMS for [M + 1]^+^ [C_26_H_22_O_2_PS]: calc.: 429.1078; found: 429.1066.

*rac-(RS,8SR,9SR,10SR)-2,8-Diphenyl-4-(4-methylphenyl)-4,4a,8a-trihydrophosphinino[2,3-b]thiopyran-5-one 8-oxide* (**13c**). Yield: 245 mg (51%), cream-colored crystals, mp = 201 °C (decomp.) (petroleum ether/CH_2_Cl_2_).

^1^H NMR (CDCl_3_): δ 2.35 (*s*, CH_3_); 3.60–362 (*m*, 1CH); 3.74 (*brs*, 1CH); 4.57 (*d*, *J* = 12.9 Hz, 1CH); 6.44 (*brs*, 1CH); 6.68 (*dd*, *J* = 37.3 Hz, *J* = 12.9 Hz, 1CH); 6.84–6.88 (*m*, 1CH); 7.16, 7.56 (*AB-signal pattern*, *J* = 7.7 Hz, 4CH_arom_); 7.32–7.39 (*m*, 5CH); 7.66–7.68 (*m*, 2CH); 7.73–7.76–(*m*, *C*H); 7.91–7.95 (*m*, 2*C*H).

^13^C{^1^H} NMR (CDCl_3_): δ 21.0 (*C*H_3_), 44.8 (*d*, ^1^*J*_C,P_ = 66 Hz, *C*(9)); 44.7 (*J*_C,P_ = 9 Hz), 48.5 (*d*, *J*_C,P_ = 4 Hz) (2d, *C*(4) and *C*(10)—assignment uncertain); 120.7, 126.7, 128.5, 128.6, 128.8, 129.1, 136.6, 138.8, 143.4 (9 signals), 128.7 (*d*, ^1^*J*_C,P_ = 104 Hz *C*_ar_), 129.5 (*d*, *J* = 12 Hz), 130.5 (^1^*J*_C,P_ = 83 Hz), 131.1 (*d*, *J* = 10 Hz), 132.2 (*d*, *J* = 7 Hz),133.7 (*d*, *J* = 2 Hz), 137.5 (*d*, *J* = 2 Hz), 191.9 (*d*, ^3^*J_C,P_* = 10 Hz, *C*=O).

^31^P NMR (CDCl_3_): δ 21.4.

IR: 3023*m*, 3011*m*, 2897*m*, 1702*s* (C=O); 1593*m*, 1501*s*, 1493*m*, 1234*m*, 1166*m*, 892*s*, 745*s*, 693*vs*.

HRMS for [M + 1]^+^ [C_27_H_24_O_2_PS]: calc.: 443.1235; found: 443.1235.

EA for C_27_H_23_O_2_PS (442.12): calc. C 73.29, H 5.24, S 7.24; found C 73.21, H 5.17, S 7.10.

*rac-(RS,8SR,9SR,10SR)-4-(4-Bromophenyl)-2,8-diphenyl-4,4a,8a-trihydrophosphinino[2,3-b]thiopyran-5-one 8-oxide* (**13d**). Yield: 310 mg (61%), yellow crystals, mp = 178 °C (decomp.) (petroleum ether/CH_2_Cl_2_).

^1^H NMR (CDCl_3_): δ 3.56‒3.59 (*m*, 1CH); 3.79 (*brs*, 1CH); 4.55 (*td*, *J* = 15.4 Hz, *J* = 2.7 Hz, 1CH); 6.36 (*d*, *J* = 2.4 Hz, 1CH); 6.69 (*dd*, *J* = 37.3 Hz, *J* = 12.9 Hz, 1CH); 6.86–6.90 (*m*, 1CH); 7.31, 7.46 (*AB-signal pattern*, *J* = 8.5 Hz, 4CH_arom_); 7.36–7.40 (m, 3CH_arom_); 7.55–7.58 (*m*, 2CH_arom_); 7.65–7.69 (*m*, 2CH_arom_); 7.75–7.77 (*m*, 1CH_arom_); 7.91–7.95 (*m*, 2CH_arom_).

^13^C{^1^H} NMR (CDCl_3_): δ 44.5 (*d*, ^1^*J*_C,P_ = 66 Hz, *C*(9)), 44.4 (*J*_C,P_ = 9 Hz), 48.4 (*J*_C,P_ = 4 Hz) (2d, *C*(4) and *C*(10)—assignment uncertain); 119.7, 120.8, 126.6, 128.6, 128.8, 131.1, 131.5, 138.7, 143.2 (9 *singlet* signals); 128.5 (*d*, ^1^*J*_C,P_ = 104 Hz, *C*_ar_) 129.5 (*d*, *J* = 12 Hz), 129.6 (*d*, ^1^*J*_C,P_ = 83 Hz, *C*(7)), 131.2 (*d*, *J* = 8 Hz), 133.8 (*d*, *J* = 3 Hz), 133.2 (*d*, *J* = 7 Hz), 139.7 (*d*, *J* = 2 Hz), 191.9 (*d*, ^3^*J*_C,P_ = 10 Hz, *C*=O).

^31^P NMR (CDCl_3_): δ 20.9.

IR: 3013*m*, 3009*m*, 2894*m*, 1692*s* (C=O); 1589*m*, 1487*s*, 1434*m*, 1183*m*, 1110*m*, 883*m*, 728*s*, 691*vs*.

HRMS for [M + 1]^+^ [C_26_H_21_BrO_2_PS]: calc.: 507.0183; found: 507.0200.

EA for C_26_H_20_BrO_2_PS (506.01): calc. C 61.55, H 3.97, S 6.32 found C 61.56, H 3.92, S 6.33.

**Attempted reaction of thiochalcone 2b with 2,3-dihydro-4*H*-phosphinin-4-one 1-oxide (5b)**: A solution containing 246 mg (1.1 mmol) of thiochalcone **2b** and 206 mg (1 mmol) of phosphinin-4-one 1-oxide **5b** dissolved in 4 mL of dry THF was heated overnight at 60 °C (about 20 h). The formation of tarry products was observed, and no defined product could be isolated from the crude reaction mixture.

**Preparation of crystals of 13a and 13b for X-ray measurement**: Suitable crystals of **13a** and **13b** were obtained by slow evaporation of the solvent from a hexane/dichloromethane solution under room conditions. Samples used for crystallization were initially purified by the PLC method directly from the crude reaction mixture. Once the crystals were formed, samples of single crystals suitable for X-ray measurements were selected using a stereomicroscope with polarized-light functionality. During the X-ray measurement, crystals were mounted on loops using dedicated oil.

## 4. Conclusions

The presented work should be regarded as a continuation of a series of our studies on the preparation and structural investigation of new P-functionalized sulfur heterocycles and *P*,*S*-heterocycles. This series is based on the utilization of thiocarbonyl compounds as universal building blocks for diverse cycloaddition reactions, showcasing the significant utility of relatively unexplored thiochalcones [40,41,42].

The current study demonstrated that hitherto unexplored, prochiral phosphinin-4-one 1-oxide **5a** undergoes *hetero*-Diels–Alder reactions with thiochalcones **2** in a regiospecific and highly stereoselective manner, thereby providing access to the previously unknown *P*,*S*-heterocycles **13**. These compounds are identified as new, fused derivatives of thiopyran and phosphinine. Both diverse phosphorus-containing heterocycles and thio-pyrans are recognized as important pharmacophores, bio-isosteres, and prodrugs [43,44,45], and therefore, the described cycloadducts **13** may be of potential interest for further investigations as biologically active compounds, particularly in medicinal chemistry and crop protection agriculture science. Additionally, the described chiral *P*,*S*-heterocycles, available in enantiopure form, may be of interest for phosphorus-based catalysis, an area undergoing dynamic development in recent decades [46].

In summary, this work complements the latest contributions on synthetically useful applications of thiochalcones [47] and underlines their significance in the development of methods for the preparation of novel, sulfur-containing heterocycles.

## Data Availability

Data are contained within the article and Supplementary Materials.

## Supplementary Materials

The following supporting information can be downloaded at: https://www.mdpi.com/article/10.3390/molecules29092036/s1: Copies of the ^1^H, ^13^C, and ^31^P NMR spectra of all new compounds **13a**–**13d,** as well as the X-ray structure determination for cycloadduct **13a**,**b**.

Figure S1. Fragment of the ^1^H NMR spectra registered for unseparated crude mixture obtained after (4+2)-cycloaddition of **2a** and **5a**. Figure S2. The ^1^H NMR spectrum for cycloadduct **6a**. Figure S3. The ^13^C NMR spectrum for cycloadduct **6a**. Figure S4. The ^31^P NMR spectrum for cycloadduct **6a**. Figure S5. The ^1^H NMR spectrum for cycloadduct **6b**. Figure S6. The ^13^C NMR spectrum of cycloadduct **6b**. Figure S7. The ^31^P NMR spectrum registered for cycloadduct **6b**. Figure S8. The ^1^H NMR spectrum registered for cycloadduct **6c**. Figure S9. The ^13^C NMR spectrum registered for cycloadduct **6c**. Figure S10. The ^31^P NMR spectrum registered for cycloadduct **6c**. Figure S11. The ^1^H NMR spectrum registered for cycloadduct **6d**. Figure S12. The ^13^C NMR spectrum registered for cycloadduct **6d.** Figure S14. Molecular structure of the 4-(thien-2-yl) substituted cycloadduct **6a**. Atoms are represented by thermal ellipsoids (50%). Figure S15. Molecular structure of the 4-phenyl substituted cycloadduct **6b**. Atoms are represented by thermal ellipsoids (50%). Table S1. Crystal data and structure refinement for **6a** and **6b**.
