# Supplementary material for: Novel Bicyclic P,S-Heterocycles via Stereoselective hetero-Diels–Alder Reactions of Thiochalcones with 1-Phenyl-4H-phosphinin-4-one 1-Oxide
Source: Molecules. 2024 Apr 28;29(9):2036. doi: 10.3390/molecules29092036 (PMC11085538; doi:10.3390/molecules29092036)
Supplement: Supplementary file 1 [file molecules-29-02036-s001.zip › molecules-2916118-supplementary.pdf]

Supplementary materials

Novel Bicyclic P,S-Heterocycles via Stereoselective hetero-Diels–Alder Reactions of Thiochalcones with 1-Phenyl-4H-phosphinin-4-one 1-Oxide †

Grzegorz Mlostoń [1,*], Katarzyna Urbaniak [1], Marcin Palusiak [2], Elżbieta Łastawiecka [3], Sławomir Frynas [3], Kazimierz Michał Pietrusiewicz [3,*] and Heinz Heimgartner [4]

1 Department of Organic and Applied Chemistry, Faculty of Chemistry, University of Lodz, Tamka 12, 91-403 Lodz, Poland; katarzyna.urbaniak@chemia.uni.lodz.pl
2 Department of Physical Chemistry, Faculty of Chemistry, University of Lodz, Pomorska 163/165, 90-236 Lodz, Poland; marcin.palusiak@chemia.uni.lodz.pl
3 Department of Organic and Crystal Chemistry, Institute of Chemical Sciences, Faculty of Chemistry, Maria Curie-Skłodowska University, Gliniana 33, 20-614 Lublin, Poland; elzbieta.lastawiecka@mail.umcs.pl (E.Ł.); slawomir.frynaslas@mail.umcs.pl (S.F.)
4 Department of Chemistry, University of Zurich, Winterthurerstrasse 190, CH-8057 Zurich, Switzerland; heinz.heimgartner@chem.uzh.ch
* Correspondence: grzegorz.mloston@chemia.uni.lodz.pl (G.M.); kazimierz.pietrusiewicz@poczta.umcs.lublin.pl (K.M.P.); Tel.: +48-42-635-57-61 (G.M.)
† In memory of Professor Jan Epsztajn (Lodz).

Content:

1. A copy of the aliphatic fragment of the 1H NMR spectrum of crude products 6a and 7a, and copies of ^{1}H NMR, ^{13}C NMR, and ^{31}P NMR spectra of all synthesized compounds,

   page 2

2. Crystal structure determinations of cycloadducts **6a** and, **6b**, page 10

1. **A copy of the aliphatic fragment of the ^{1}H NMR spectrum of crude products 6a and 7a, and copies of ^{1}H NMR, ^{13}C NMR, *and* ^{31}P NMR spectra of isolated cycloadducts 6a–6d**

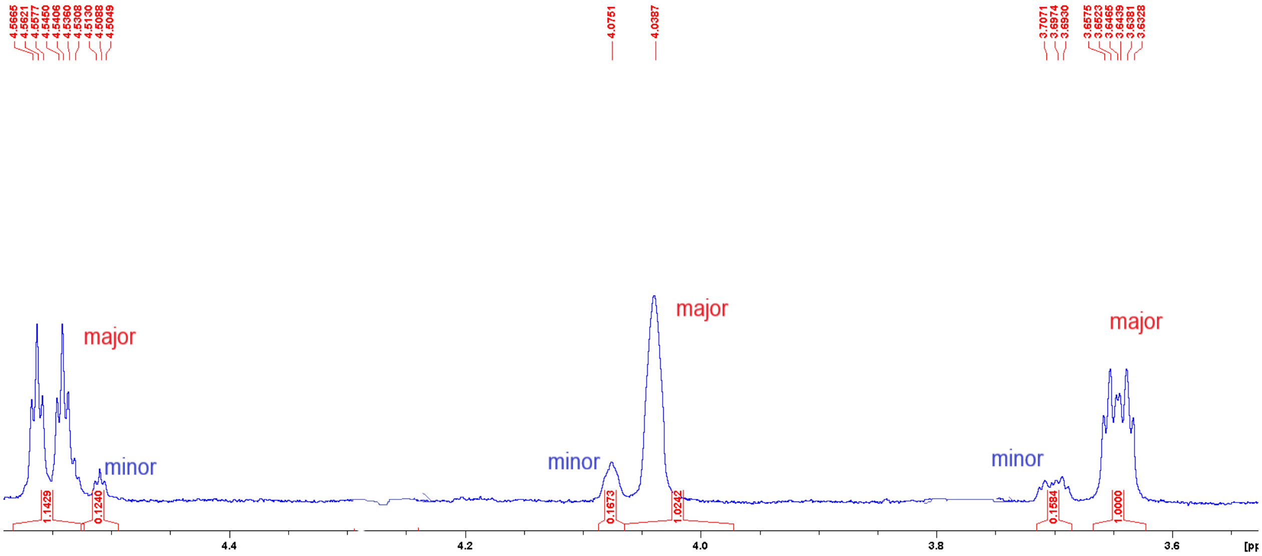

Figure S1. Fragment of the ^{1}H NMR spectra registered for unseparated crude mixture obtained after (4+2)-cycloaddition of **2a** and **5a**.

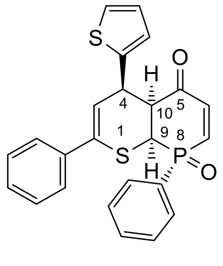

6a

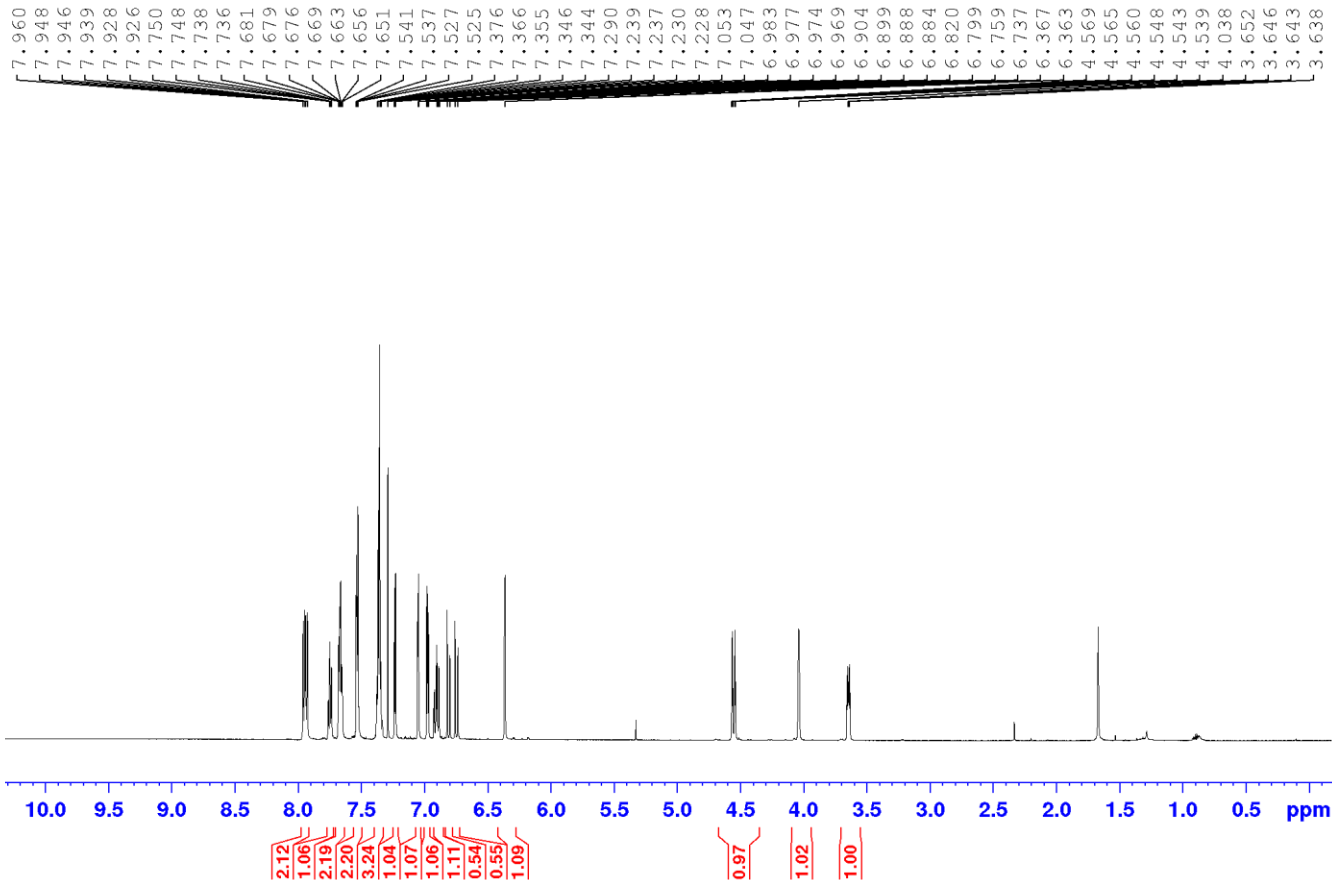

Figure S2. The ^{1}H NMR spectrum for cycloadduct **6a**.

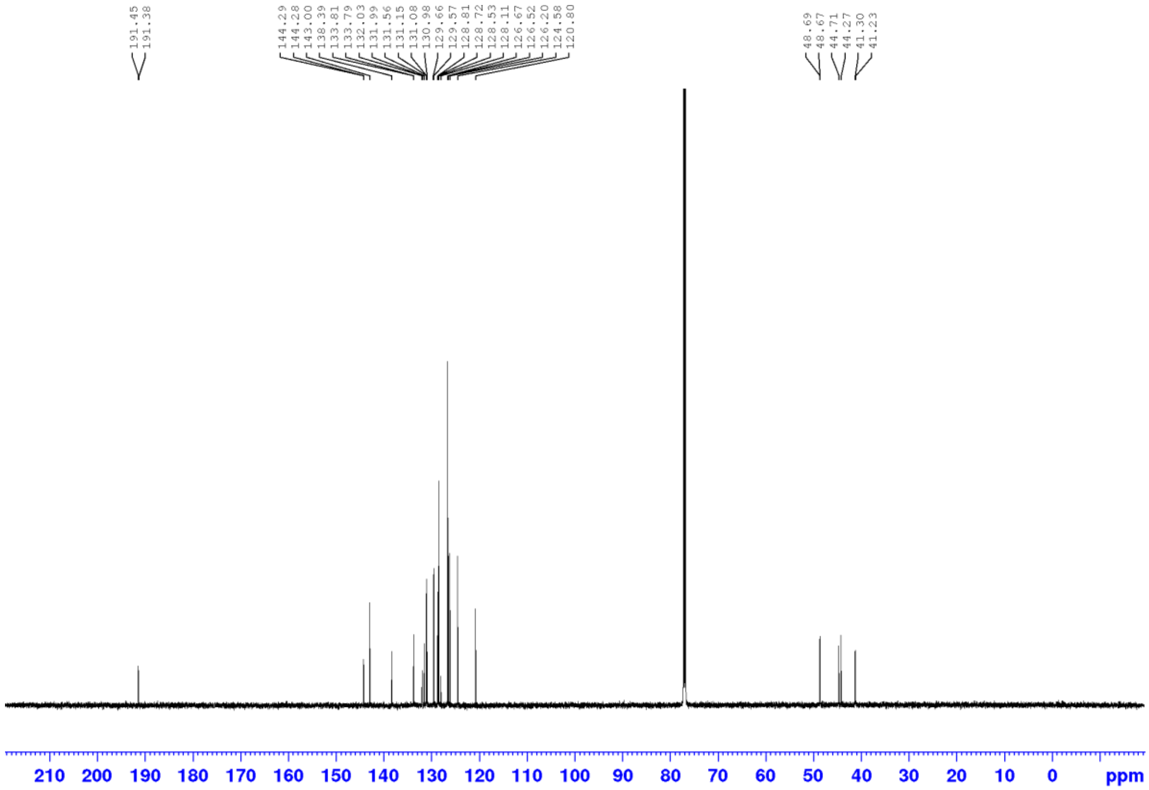

Figure S3. The ^{13}C NMR spectrum for cycloadduct **6a**.

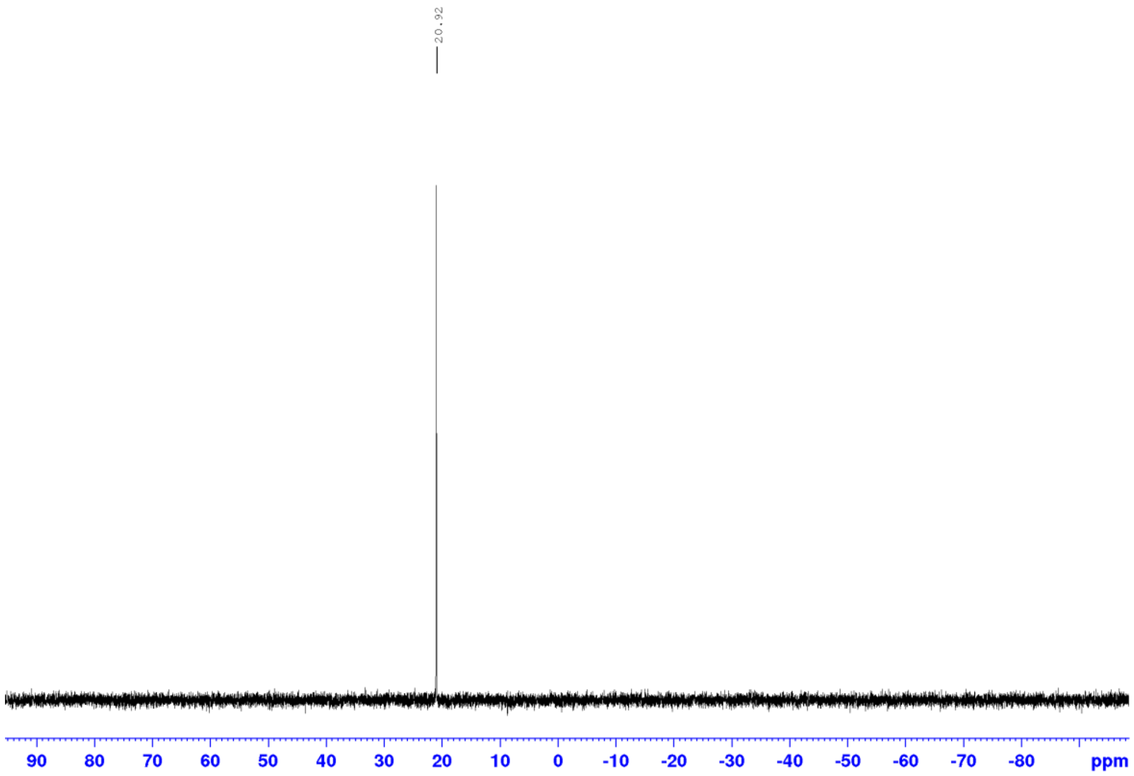

Figure S4. The ^{31}P NMR spectrum for cycloadduct **6a**.

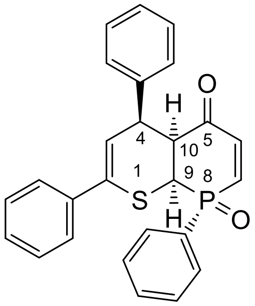

6b

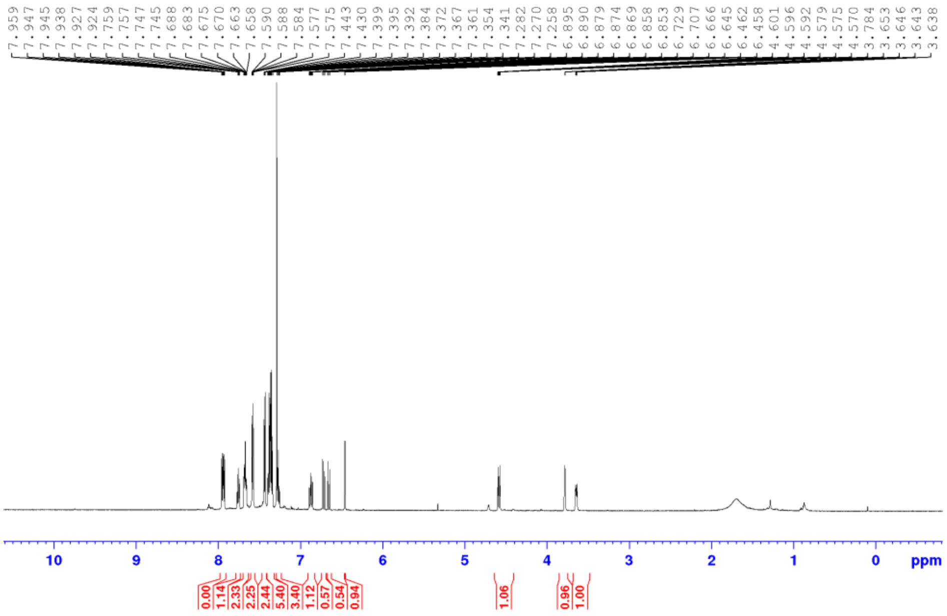

Figure S5. The ^{1}H NMR spectrum for cycloadduct **6b**.

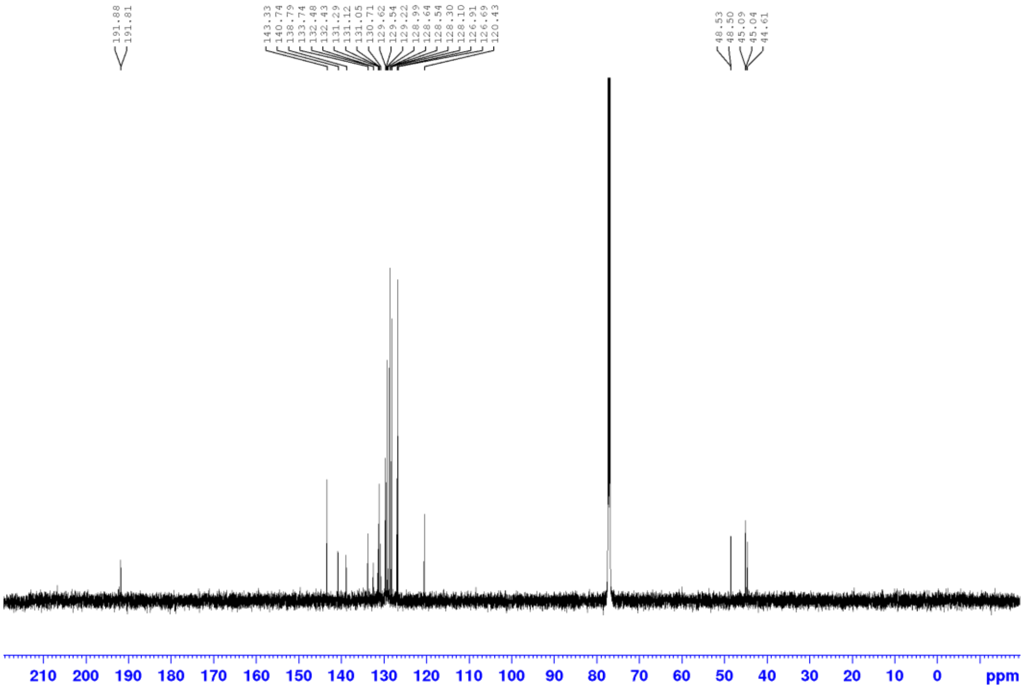

Figure S6. The ^{13}C NMR spectrum of cycloadduct **6b**.

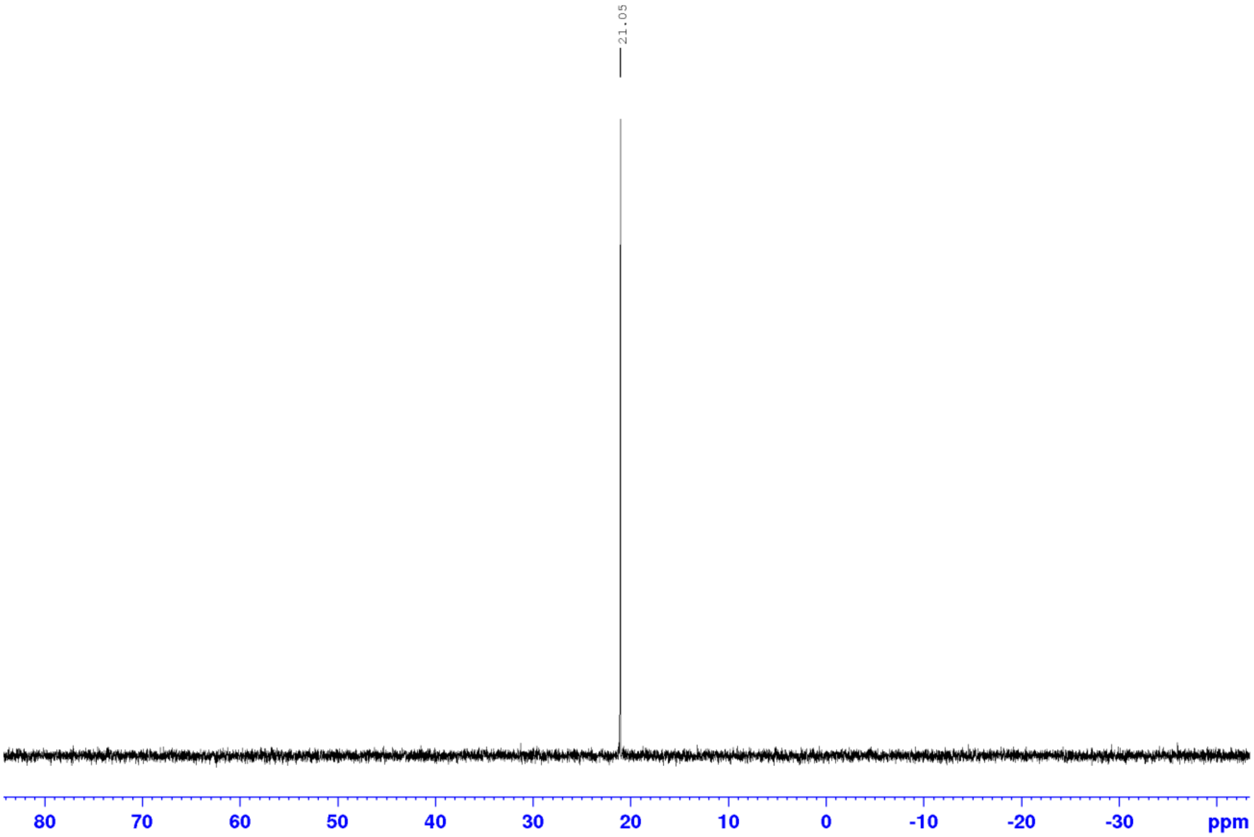

Figure S7. The ^{31}P NMR spectrum registered for cycloadduct **6b**.

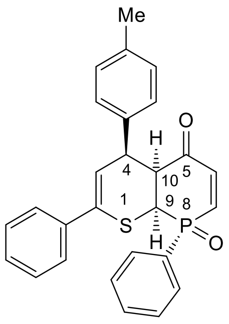

6c

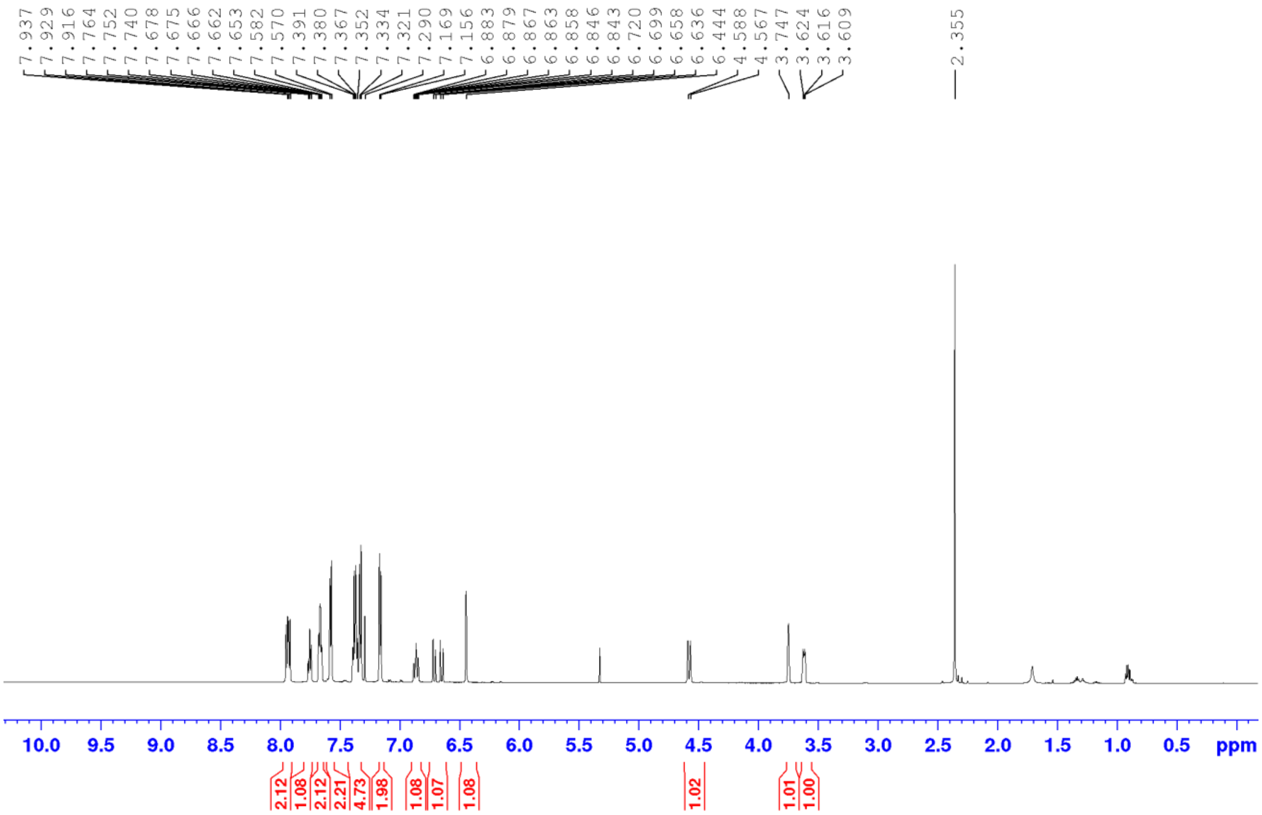

Figure S8. The ^{1}H NMR spectrum registered for cycloadduct **6c**.

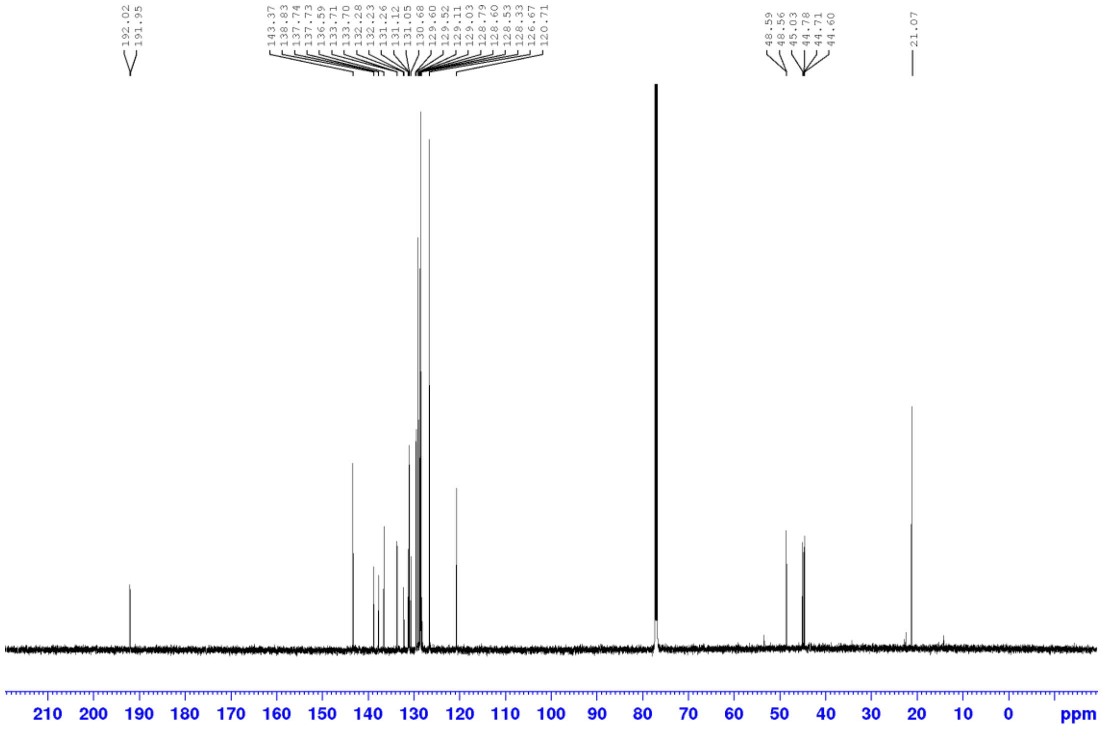

Figure S9. The ^{13}C NMR spectrum registered for cycloadduct **6c**.

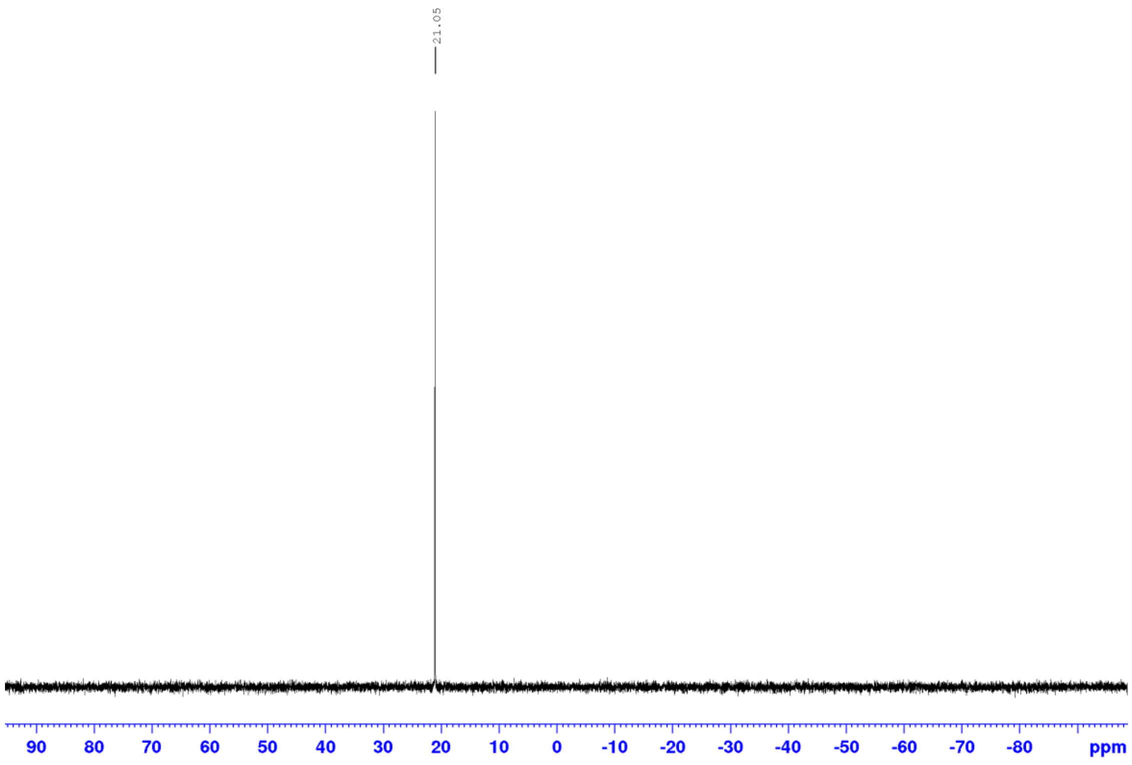

Figure S10. The ^{31}P NMR spectrum registered for cycloadduct **6c**.

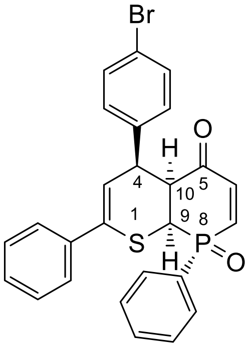

6d

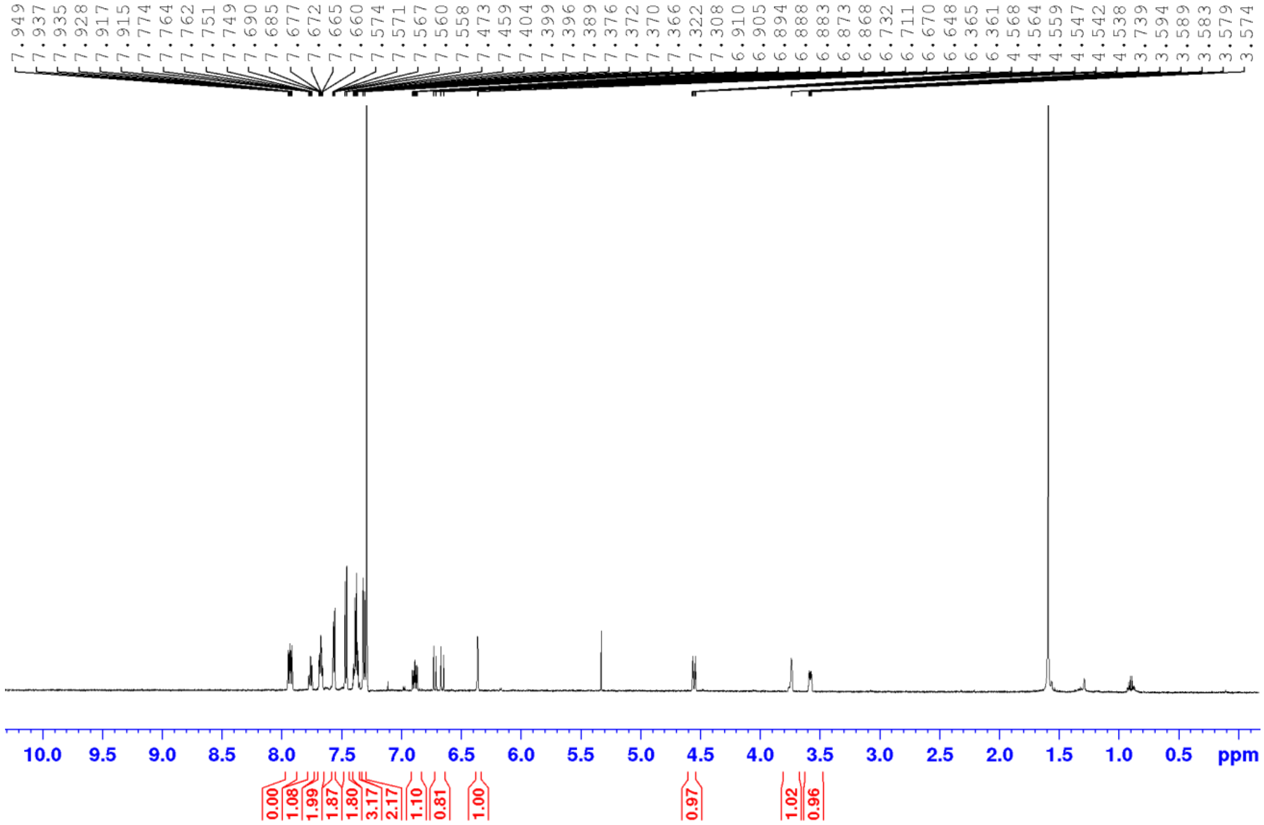

Figure S11. The ^{1}H NMR spectrum registered for cycloadduct **6d**.

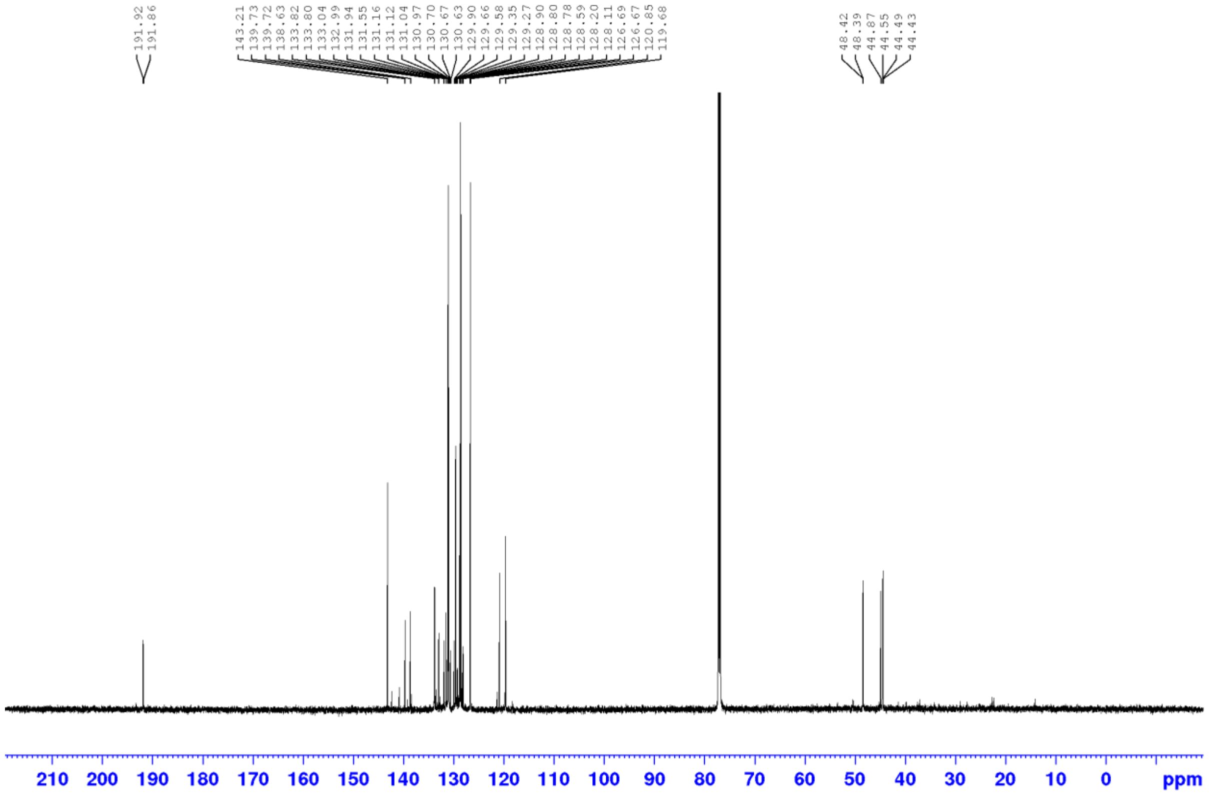

Figure S12. The ^{13}C NMR spectrum registered for cycloadduct **6d**.

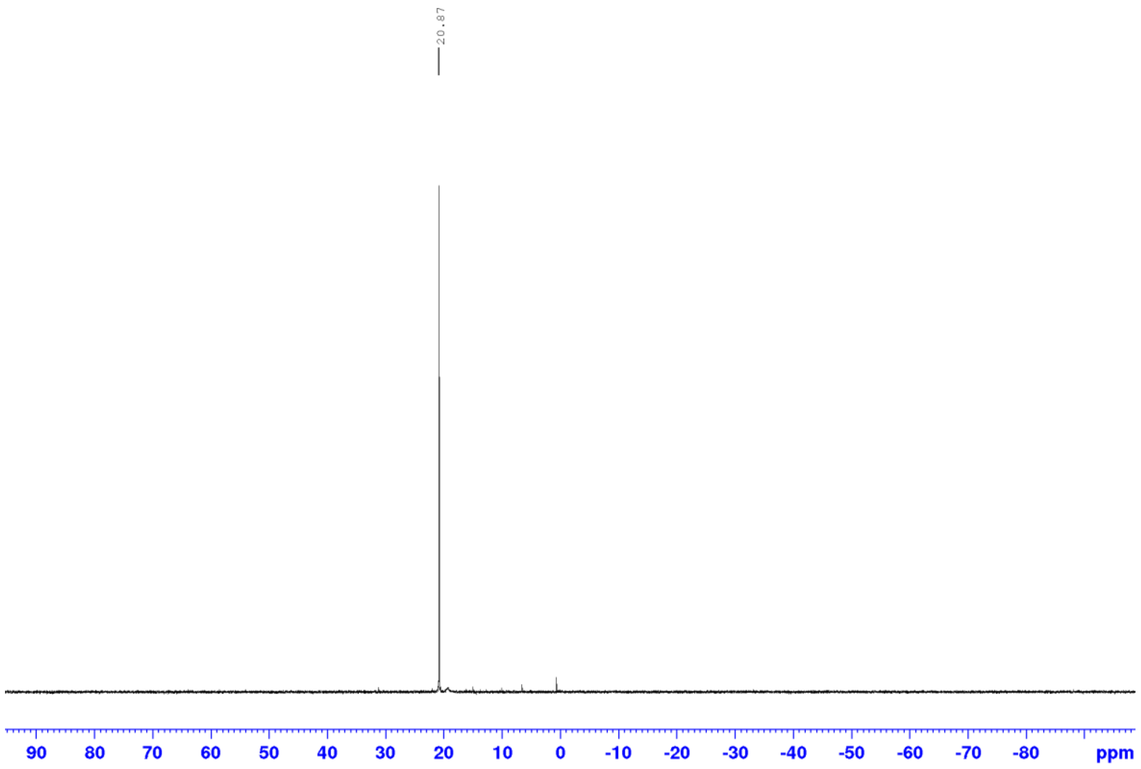

Figure S13. The ^{31}P NMR spectrum registered for cycloadduct **6d**.

2. Crystal structure determinations of cycloadducts 6a and 6b.

Experimental: X-Ray structure determination of **6a** and **6b**.

Generals : X-ray diffraction data for **6a** and 6b was collected on an XtaLAB Synergy, Dualflex, HyPix diffractometer. Integration of the intensities and corrections for Lorentz effects, polarization effects, and analytical absorption were performed with CrysAlis PRO [1]. Using Olex2 [2], the structure was solved with the SHELXT [3] structure solution program using Intrinsic Phasing and refined with the SHELXL [4] refinement package using Least Squares minimization. The hydrogen atoms were introduced in the calculated positions with an idealized geometry and constrained using a rigid body model with isotropic displacement parameters equal to 1.2 of the equivalent displacement parameters of their parent atoms. The molecular geometries were calculated by the PLATON program [5]. The relevant crystallographic data are given in Table S1 (SI). Atomic coordinates, displacement parameters, and structural factors of the analyzed crystal structures are deposited with the Cambridge Crystallographic Data Centre CCDC (reference number: 2298978 and 2298979) [6].

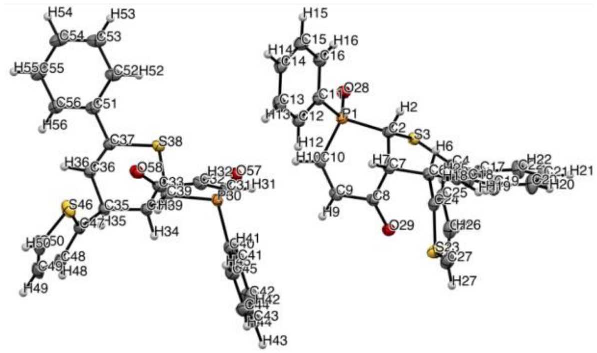

Figure S14. Molecular structure of the 4-(thien-2-yl) substituted cycloadduct **6a**. Atoms are represented by thermal ellipsoids (50%).

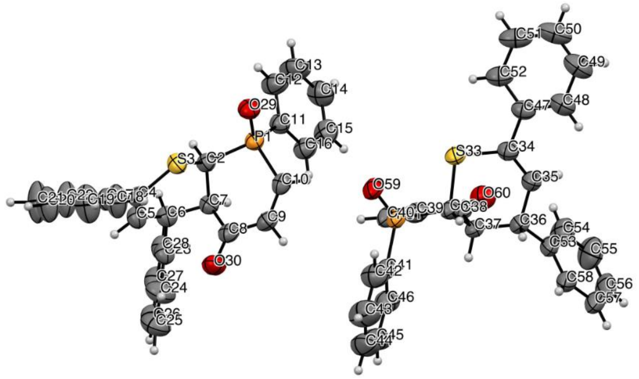

Figure S15. Molecular structure of the 4-phenyl substituted cycloadduct **6b**. Atoms are represented by thermal ellipsoids (50%).

Table S1. Crystal data and structure refinement for **6a** and **6b.**

Identification code	Compound **6a**	Compound **6b**
Empirical formula	$C_{24}H_{19}O_2PS_2$	$C_{26}H_{21}O_2PS$
Formula weight	434.48	428.45
Temperature/K	99.99(16)	293(2)
Crystal system	monoclinic	monoclinic
Space group	$P2_1/c$	$P2_1/c$
a/Å	17.8452(2)	18.1850(2)
b/Å	21.0134(3)	21.3321(2)
c/Å	11.48980(10)	11.69800(10)
α/°	90	90
β/°	105.5390(10)	106.8580(10)
γ/°	90	90
Volume/Å^3	4151.06(9)	4342.93(8)
Z	8	8
ρ_{calc}mg/mm^3	1.390	1.311
μ/mm^{-1}	3.199	2.175
F(000)	1808.0	1792.0
X-ray source	Cu Kα (λ = 1.54184)	Cu Kα (λ = 1.54184)
2Θ range for data collection	5.14 to 153.214	5.078 to 153.186°
Index ranges	$-19 \le h \le 22$, $-25 \le k \le 26$, $-14 \le l \le 11$	$-22 \le h \le 20$, $-26 \le k \le 26$, $-13 \le l \le 14$
Reflections collected	39227	95769
Independent reflections	8450 [R_{int} = 0.0502]	8934[R_{int} = 0.0608]
Data/restraints/parameters	8450/0/523	8934/0/541
Goodness-of-fit on F^2	1.065	1.038
Final R indexes [I>=2σ (I)]	R_1 = 0.0375, wR_2 = 0.1005	R_1 = 0.0405, wR_2 = 0.1096
Final R indexes [all data]	R_1 = 0.0409, wR_2 = 0.1031	R_1 = 0.0469, wR_2 = 0.1138
Largest diff. peak/hole / e Å^{-3}	0.46/-0.38	0.43/-0.28

CCDC number	2331463	2331462

References:

1. CrysAlisPRO software system, Oxford Diffraction/Agilent Technologies UK Ltd, Yarnton, England, 2015.
2. O.V. Dolomanov, L.J. Bourhis, R.J. Gildea, J.A.K. Howard, H. Puschmann, OLEX2: A Complete Structure Solution, Refinement and Analysis Program. *J. Appl. Crystallogr.* **2009**, *42*, 339–341.
3. G.M. Sheldrick, SHELXT - Integrated space-group and crystal-structure determination. *Acta Cryst. Sect. A: Foundations and Advances* **2015**, *71*, 3–8.
4. G. M. Sheldrick, Crystal structure refinement with SHELXL. *Acta Crystallogr. Sect. C: Struct. Chem*. **2015**, *71*, 3–8.
5. A. L. Spek, Structure validation in chemical crystallography. *Acta Crystallogr. Sect. D: Biol. Crystallogr*. **2009**, *65*, 148–155.
6. C. R. Groom, I. J. Bruno, M. P. Lightfoot and S. C. Ward, The Cambridge Structural Database, *Acta Cryst*. **2016**, *B72*, 171–179.
